# Metabolic Aspects of Migraine: Association With Obesity and Diabetes Mellitus

**DOI:** 10.3389/fneur.2021.686398

**Published:** 2021-06-09

**Authors:** Eduardo Rivera-Mancilla, Linda Al-Hassany, Carlos M. Villalón, Antoinette MaassenVanDenBrink

**Affiliations:** ^1^Division of Vascular Medicine and Pharmacology, Department of Internal Medicine, Erasmus University Medical Center, Rotterdam, Netherlands; ^2^Department of Pharmacobiology, Cinvestav-Coapa, Mexico City, Mexico

**Keywords:** CGRP, comorbidities, diabetes mellitus, lifestyle, metabolic disorders, migraine, obesity

## Abstract

Migraine is a disabling neurovascular disorder, characterized by moderate to severe unilateral headaches, nausea, photophobia, and/or phonophobia, with a higher prevalence in women than in men, which can drastically affect the quality of life of migraine patients. In addition, this chronic disorder is related with metabolic comorbidities associated with the patient's lifestyle, including obesity and diabetes mellitus (DM). Beyond the personal and socioeconomic impact caused by migraine, obesity and DM, it has been suggested that these metabolic disorders seem to be related to migraine since: (i) they are a risk factor for developing cardiovascular disorders or chronic diseases; (ii) they can be influenced by genetic and environmental risk factors; and (iii) while clinical and epidemiological studies suggest that obesity is a risk factor for migraine, DM (i.e., type 1 and type 2 DM) have been reported to be either a protective or a risk factor in migraine. On this basis, and given the high worldwide prevalence of migraine, obesity, and DM, this article provides a narrative review of the current literature related to the association between the etiology and pathophysiology of migraine and these metabolic disorders, considering lifestyle aspects, as well as the possible involvement of neurotransmitters, neuropeptides, and/or sex hormones. While a link between migraine and metabolic disorders has been suggested, many studies are contradictory and the mechanisms involved in this association are not yet sufficiently established. Therefore, further research should be focused on understanding the possible mechanisms involved.

## Introduction

Migraine is a disabling neurovascular disorder three times more prevalent in women than in men (1). It is the second-highest specific cause of disability, and the first in those individuals under 50 years of age (2, 3). Migraine attacks are characterized by recurrent moderate to severe headaches accompanied by nausea, vomiting, phonophobia and/or photophobia (2, 4). Besides affecting the quality life of migraine patients, it has a dramatic social and economic impact (3).

**Figure 1:**
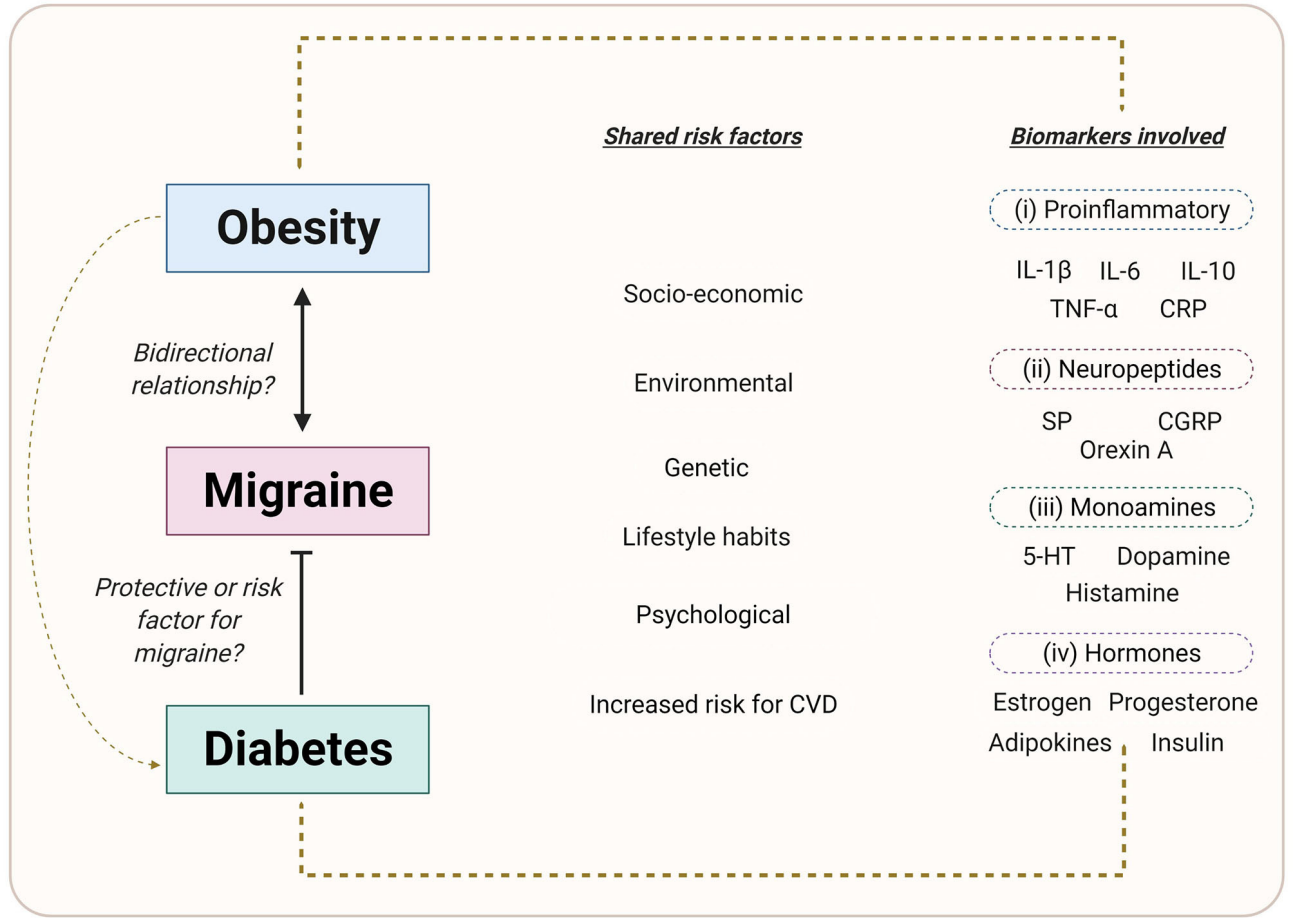
Common factors between migraine, obesity and diabetes mellitus. Environmental, genetic, and lifestyle factors are involved in the etiology of migraine, obesity, and DM. In addition, the role of pro-inflammatory markers, neuropeptides, adipokines, sex hormones, and/or monoamines in the onset, development and progression of the disease, may indicate a possible link between these disorders. Created with BioRender.com.

In the last years, it has become evident that migraine is associated with several comorbidities including cardiovascular diseases, pain disorders, psychiatric, or neurological comorbidities (5, 6). Furthermore, a direct and/or indirect link between migraine attacks and metabolic/endocrine disorders (i.e., obesity and diabetes mellitus, DM) has recently been described (7–13); which could be related to patient's lifestyle habits. In addition, some mechanisms can be hypothesized to suggest a link between migraine and metabolic/endocrine disorders (Figure 1), including: (i) the risk factor to develop cardiovascular complications (14, 15); (ii) the influence of socioeconomic, environmental, genetic and/or psychological factors (7, 12, 16, 17); and (iii) the involvement of biochemical biomarkers, including neuropeptides (8, 18–26), pro-inflammatory mediators (27–36), or adipokines (37–39) (Table 1), which play an important role in the pathophysiology of migraine (e.g., calcitonin gene-related peptide, CGRP).

**Table 1:** Relevant biochemical biomarkers in migraine, obesity, and diabetes mellitus (DM).

**Biochemical biomarker**	**Migraine**	**Obesity**	**DM**	**References**
**Pro-inflammatory**				
C-reactive protein	Increased both in migraine with and without aura	Increased	Increased in both type 1 and type 2 DM	(29, 30, 33, 34)
IL-1β	Higher levels during migraine attacks, both in migraine with and without aura	Increased in morbidly obese patients	Higher levels in both type 1 and type 2 DM	(27, 31, 35, 36)
IL-6	No differences in patients outside and during migraine attacks	Increased	Elevated in both poorly and controlled DM	(27, 30, 33)
IL-8	Increased interictal levels in migraine patients	Increased levels and BIM-related	Elevated older patients with DM	(28, 30, 32)
TNF-α	Higher levels during migraine attacks, in both migraine with and without aura	Higher concentrations	Elevated levels	(27, 30, 33)
**Neuropeptides**				
CGRP	Higher levels both in migraine with and without aura	Higher levels	Decreased in type 2 DM	(18, 19, 21, 24)
Substance P	Higher levels both in migraine with and without aura	Higher levels in obese patients	Decreased in type 2 DM	(19, 22, 24)
Neuropeptide Y	Tended to increase during migraine attacks in migraine with aura	Increased	Increased in type 2 DM	(20, 25, 26)
**Adipokines**				
Leptin	Higher levels in migraine with aura	Higher concentrations	Increased in type 2 DM	(37–39)
Adiponectin	Increased levels in both episodic and chronic migraine	Decreased levels	Decreased in type 2 but increased in type 1 DM	(37, 40–43)

Both obesity and DM are chronic metabolic and lifestyle-related disorders with a high worldwide prevalence (44). Moreover, obesity has been suggested to be a promoter of either type 1 or type 2 DM (Figure 1) (44–46), increasing the risk to develop comorbidities. The question remains open whether migraine can be considered, at least partly, a metabolic/endocrine disorder, and how it is associated with obesity and DM. In this respect, several studies have shown that there is a complex and controversial association between these three disorders (7, 9–13, 16, 17). On the one hand, several studies indicate an association between obesity and chronic daily headache, including chronic tension-type headache and chronic migraine (47, 48), as well as migraine with aura (49). Apart from a direct comorbidity, obesity has been suggested to be associated with the frequency and severity of migraine attacks (50). In addition, animal studies have provided a possible link between obesity and migraine, since several vascular mediators (e.g., CGRP), pro-inflammatory biomarkers (e.g., interleukins, cytokines), or substances involved in the food intake and weight control (e.g., orexin) have an important role in both obesity and migraine (51–53) (see Figure 1). On the other hand, findings on the link between DM and migraine are controversial. In this respect, clinical- as well as population-based studies have reported that the prevalence of migraine in diabetic patients is lower (54–57), similar (58–60) or higher (61) when compared with non-diabetic patients. Furthermore, several authors have suggested that DM can be a protective factor in migraine (13, 57, 62).

Accordingly, and given the clinical relevance besides the high worldwide prevalence of migraine, obesity, and DM, this review attempts to provide a general overview of the association between migraine and metabolic disorders related to the patient's lifestyle, considering: (i) both etiology and pathophysiology; (ii) evidence from clinical and epidemiological studies; and (iii) the possible underlying mechanisms. Nevertheless, we consider that further basic experimental science and clinical or epidemiological research should be focused on clarifying controversial results in order to improve the understanding of the relationship between these disorders, which could subsequently help to develop therapies to improve the severity or frequency of headache attacks in obese and/or diabetic patients with migraine.

We performed a search strategy on the 4th of January 2021 using the following databases: Embase (via: *Embase.com*), Medline ALL (via: *Ovid*), Web of Science Core Collection (via: *Web of Knowledge*), Cochrane Central Register of Controlled Trials (via: *Wiley*), and Google Scholar. The following main keywords were used: migraine with aura, migraine without aura, obesity, DM, and CGRP. Besides, reference lists of included articles were reviewed to find articles that were not yet retrieved. No restriction in the search period was applied to be as complete as possible. Details of the search strategy can be found in Appendix 1.

## Migraine, Obesity, and Diabetes Mellitus: an Overview

Globally, migraine, obesity, and DM represent three major public health problems in all age groups and in both sexes. Furthermore, obesity (63) and DM (64) are considered “a pandemic” due to increases in their prevalence. In this respect, migraine represents the second-highest specific cause of disability, and the first in those people under 50 years of age (2, 3) with an age-standardized prevalence of 14.4% (18.9% for women and 9.8% for men) (3). Moreover, obesity has an estimated prevalence of 13.0% in adult populatio (15.0% for women and 11.0% for men) (65); while DM represents an estimated prevalence of 9.3% (9.0% for women and 9.6% for men) (66). Certainly, classical mechanisms in the pathogenesis and pathophysiology of migraine, obesity, and DM have been described. Nevertheless, it has been suggested that lifestyle and/or genetic and environmental factors may contribute to the development of these disorders (7, 9, 67–74).

### Etiology and Pathophysiology of Migraine

Migraine is clinically characterized by several symptoms, including nausea, vomiting, phonophobia, and/or photophobia (2, 4). According to the number of headache days per month, migraine has been classified by the International Headache Society (2) into: episodic migraine –characterized by presenting <15 headache days per month; and chronic migraine—characterized by presenting ≥15 headache days per month during more than 3 months with experiencing migraine features in at least 8 days per month (2).

The mechanisms contributing to the pathogenesis of migraine are still poorly understood and very debated. Over time, the main or “classical” driving and/or generating mechanisms of migraine have been described, including: (i) hypothalamic and brainstem activation, which is involved in starting, maintaining, an ending the migraine attacks (75–77); (ii) cortical spreading depression, the underlying mechanism involved in migraine aura's (77, 78); and (iii) activation of the trigeminovascular system and the release of CGRP from sensory nerves, involved in the development of headache and migraine-related symptoms (79–81). Most recently, findings from neuroimaging and neurophysiological studies have demonstrated that occipital cortices, which are involved during an aura, are characterized by an abnormal metabolism in migraine. In addition, fluctuations in the excitability (including imbalanced inhibitory and excitatory circuits of the cortex) and plasticity are thought to play a role (82). Moreover, genetic factors have been described to contribute to an individual's susceptibility to migraine (83). In this respect, hemiplegic migraine, for instance, is an autosomal dominant disorder, which is linked to the genes CACNA1A, ATP1A2, and SCN1A that encode ion transport proteins or ion channels (83, 84). To our knowledge, nevertheless, an association between hemiplegic migraine with obesity and DM has not been described. However, it has been reported that this type of migraine shows comorbidity with various disorders, such as epilepsy, depression, as well as sleep disorders and vascular disorders (85–88), which also represent risk factors for migraine attacks, obesity, and DM. Besides, since there are polymorphisms in the CACNA1A and ATP1A2 genes in both obesity (89, 90) and DM (91–93), we can hypothesize that genetic polymorphisms might be a possible link between these disorders. Admittedly, further experiments should be performed using genetic murine models, which also share similarities with some comorbidities associated with migraine (i.e., cardiovascular disorders) (94) in order to elucidate the possible relationship between genetic polymorphisms, migraine, obesity, and DM.

Furthermore, lifestyle habits, genetic, and environmental factors including inconsistencies in diet, caffeine intake, amount of sleep, as well as smoking or drug usage can be precipitating factors in migraine (7, 9, 67–69).

### Etiology and Pathophysiology of Obesity

Obesity is a clinical disorder with a high worldwide prevalence, and personal and social impact. This condition has been defined as abnormal or excessive fat accumulation that affects health and the quality of life of individuals (65, 95), and is characterized by presenting a body mass index (a simple index of weight-for-height used to classify overweight and obesity in adults, BMI) of ≥30 kg/m^2^ (65).

The onset, development, and progression of obesity depends on genetic, environmental, and lifestyle factors (Figure 1), including physical inactivity, dietary habits, nutrient intakes, and metabolic factors (65, 71, 72). In addition, central and peripheral mechanisms are involved in the regulation of metabolism and food intake. In this respect, the hypothalamus plays a vital role in the central mechanisms via the release of hypothalamic neuropeptides such as neuropeptide Y or orexin for the regulation of both energy intake and energy expenditure (96, 97), while the sympathetic nervous system and the release of adipokines such as leptin and adiponectin, are involved in peripheral mechanisms (96–98). Therefore, an imbalance in the regulation of metabolism or in the anatomical structures involved can trigger obesity.

### Etiology and Pathophysiology of DM

DM is a group of metabolic diseases characterized by chronic hyperglycemia with alterations in the metabolism of carbohydrates, lipids and proteins; resulting from defects in both the secretion or action of insulin (99, 100). According to the American Diabetes Association (100), DM has been classified into type I, caused by a deficiency in insulin secretion which involves the autoimmune destruction of pancreatic β cells with consequent insulin deficiency, and type II, caused by either a combination of resistance or an abnormal action of insulin. Further forms of DM include other specific types of DM, mainly characterized by genetic defects; and gestational DM, caused by hyperglycemia during pregnancy. Furthermore, like migraine and obesity, DM can also be triggered by the influence of lifestyle-related habits and genetic and environmental factors (73, 74). In addition, a dysfunction in the autonomic nervous system plus physical inactivity may be associated with a dysregulation in glucose metabolisms and the development of DM (101). Hence, an improvement in lifestyle could improve the quality of life of diabetic patients, and even reduce the appearance of DM-related complications (e.g., cardiovascular risk) (73, 74).

## Association of Migraine and Obesity

**Figure 2:**
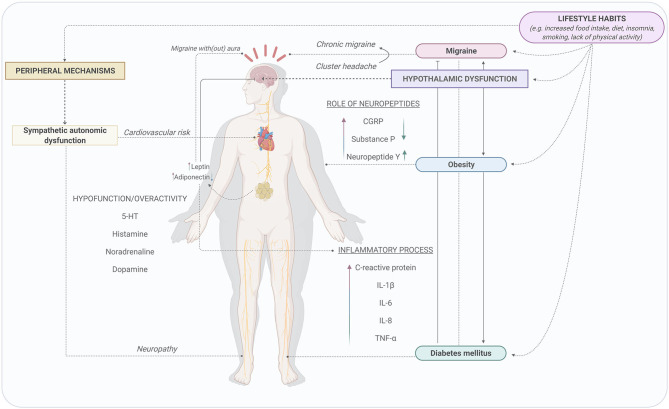
Possible mechanisms involved in the association between migraine, obesity, and diabetes mellitus. Lifestyle habits can modify the activity of the central and peripheral nervous systems. The influence of the central nervous system via the activation of hypothalamus and the sympathetic autonomic dysfunction during migraine attacks, obesity, and DM might be potential mechanisms to describe the relationship between migraine and these metabolic disorders. Created with BioRender.com.

Although several hypotheses have been postulated during the last few years, the association between migraine and obesity remains unclear. Certainly, obesity may be a determining factor in triggering more severe migraine attacks (7). Nevertheless, there are a number of lifestyle factors that may be important confounders factors which may explain the possible relationship between migraine and obesity, including smoking, indoor and outdoor air pollution, physical activity level, altitude, blood pressure, stress, rest and sleep, even drug usage (9, 102–106). Furthermore, a relation between migraine and obesity is conceivable, as several biochemical biomarkers (7) and the influence of central and peripheral mechanisms (107) are involved in the pathophysiology of both obesity and migraine (Table 1, Figure 2).

### Clinical and Epidemiological Evidence

In view of the increasing prevalence of obesity, the (direct) association and overlap between migraine and obesity has been studied for approximately the last 20 years. Chai et al. systematically summarized results from population-based studies published between 2000 and 2013 on this association (108). Most studies in this systematic review were performed in individuals with a mean age below fifty years—an age category which is predominantly affected by migraine (109)—and confirm, in general, a significant association between obesity and both episodic and chronic migraine (108). In addition, the risk of migraine seems to increase with increasing obesity status. No associations were found in perimenopausal or in postmenopausal women (108). Besides, an increased risk of having migraine was found both in underweight individuals and in obese women, but not in obese men, compared to individuals with a normal weight (106).

This association between obesity and the prevalence and frequency of migraine has also been confirmed by a recently published population-based study by Kristoffersen et al. (110). Indeed, a dose-response relationship between obesity categories—more specifically abdominal obesity—and migraine attack frequency was found (110). Moreover, a meta-analysis by Gelaye et al., including a total amount of 288,981 unique individuals from 12 studies, also showed the increased risk of migraine in participants with underweight and obesity, although the former association seemed to be less profound. These associations only remained after adjusting for age and sex (111). According to a systematic review by Farello et al., albeit investigated in very few studies, a higher prevalence of migraine was also reported in obese and overweight children (112). Research on the reverse direction—the risk of obesity in migraine patients—has been performed to a much lesser extent. A pooled analysis of two studies did not result in a significant risk of obesity in migraine sufferers (106).

More recently, studies have been performed to investigate the relationship between migraine characteristics and obesity. Results from the American Migraine Prevalence and Prevention (AMPP) study already showed an increase of the migraine headache frequency as a function of the BMI, not only in subjects with migraine, but to a lesser extent also in subjects suffering from probable migraine (113). Within the same longitudinal survey, it was found that obesity was significantly more often reported in individuals suffering from chronic migraine, compared to individuals suffering from episodic migraine (25.5 vs. 21.0%, respectively, OR 1.2, 95% [1.03–1.50]) (114). These results are in accordance with a cross-sectional study in female migraine patients by Togha et al., showing that overweight and obese migraine patients (with a BMI ≥25) experience a higher frequency, severity, duration, and disability of their headache attacks, compared to those with a BMI <25 (115). Almost similar results were found in earlier cross-sectional studies including Indian and Iranian populations (116, 117). Indeed, the latter study reports a significant association between abdominal obesity and the severity, frequency, and headache diary results, except the duration of attacks, in both sexes (both after pooled and stratified analyses) (117). Kristoffersen et al. also report an association of both total body obesity and abdominal obesity with migraine prevalence as well as attack frequency (110).

However, it should be noted that these findings have not been reproduced by all studies. Mattsson did not find a significant association between migraine (characteristics) and obesity in women aged 40–74 years (118). Consistent with these findings, Winter et al. analyzed cross-sectional data from three different cohorts in Germany and showed no significant age- and gender-adjusted associations between complete migraine prevalence and BMI. The same results were observed for the prevalence of probable migraine (119). Authors from the same research group demonstrated in an earlier cross-sectional study among women in the Women's Healthy Study that, after controlling for potential confounders, the association between the increased risk of having migraine and high BMI values disappeared (120). Lastly, a cross-sectional study by Bigal et al. did not result in associations between migraine prevalence and BMI, implying an absence of comorbidity—also after stratification for sex. However, the authors did find an association between BMI and certain migraine features, which included the headache frequency, severity (as well as worsening after physical activity and disability related to migraine), and the number of patients who experienced photophobia and phonophobia in more than half of their migraine attacks (50).

Table 2 contains an overview of the most important systematic reviews (and meta-analyses) examining the association between obesity and migraine.

**Table 2:** An overview of published reviews that evaluate the association between migraine and obesity.

**Article**	**(Type of) study**	**Main focus**	**Main findings on the association with migraine**
Chai et al. (108)	Systematic review	General overview	• Obese subjects have an increased risk of both episodic and chronic migraine. • The association has mainly been observed in populations of reproductive age (mean age <50, which is in line with the age when migraine is most prevalent). • No association has been observed in subjects of perireproductive or postreproductive age.
Ornello et al. (106)	Systematic review and meta-analysis of observational studies	General overview	• Compared to subjects with a normal weight, obese subjects have an increased risk to have chronic migraine. • Compared to subjects without migraine, subjects with migraine have no increased risk to be obese. • Age, attack frequency and (female) gender might be effect-modifiers in this association.
Farello et al. (112)	Systematic review	Association between obesity and migraine in children	• Compared to lean controls, obese children suffering from migraine have a higher frequency and severity of their headache attacks. • A higher obesity prevalence is observed in children with migraine than in the general population.
Pavlovic et al. (121)	Narrative review	Effects of obesity on females with migraine of various ages	• The association between migraine and obesity is mainly observed in women of reproductive age; no relation is observed in women >55 years. • Age-related and hormonal changes play a role in this relationship.

#### Lifestyle Interventions and Treatments in Migraine and Obesity

Migraine (in particular attack frequency) and obesity are both—to a certain extent—modifiable factors, either through pharmacological treatments, or through lifestyle modifications. However, it should be noted that the complexity and mechanisms of these interrelationships are still poorly understood (122).

In this respect, the effects of antimigraine drugs on body weight, as well as obesity treatments in migraine have been investigated. On the one hand, many treatments used in the preventive management of migraine, including antidepressants, antiepileptic drugs, and Ca^2+^ blockers, seem to cause weight gain as a side-effect (123). The only approved migraine treatment which has been shown to decrease appetite and stimulate weight loss is topiramate (122, 124). According to a prospective 4-month study, 37% of the migraine patients lost weight after receiving topiramate; while a high initial BMI did not predict weight loss in these migraine patients (125). Furthermore, an explorative study conducted in healthy female migraine sufferers aged 25-55 years demonstrated that, besides body weight, fat mass and BMI were also significantly reduced, in contrast to lean body mass, which was preserved (126). Therefore, these results indicate that weight loss—which was mainly achieved in the first 3 months of topiramate treatment—can be mainly attributed to a reduction of body fat mass. However, it should be noted that this study only included non-diabetic patients with a lower BMI at baseline. Therefore, these results might not be generalizable to patients with a higher BMI (126). These results are in line with findings from a small observational study by Schütt et al., which included six migraine patients without diabetes who received treatment with topiramate for 20 weeks. Results of this study indicated that weight loss was primarily caused by a decrease of visceral fat. Also, this decrease was accompanied by a decrease of plasma leptin, an increase of adiponectin and an improvement of the insulin sensitivity (127).

On the other hand, the association between migraine and obesity might have clinical consequences, as body weight management might contribute to migraine prevention. We recommend two recently published systematic reviews and meta-analyses by Di Vincenzo et al. and Dang et al., which evaluated the effects of weight loss after bariatric surgery (and behavioral interventions, including diets) on migraine characteristics (128, 129). It was shown that migraine frequency, disability, severity of the headache pain (128, 129), and attack duration were all significantly reduced after bariatric surgery (129). Behavioral interventions were shown to have similar significant effects as surgery on these migraine characteristics, except for attack duration, in both adult and pediatric populations (129). The meta-regression analysis showed that this effect of weight loss was neither correlated with obesity at baseline nor with the degree of weight loss (129).

However, both reviews and meta-analyses are mainly restricted by their relatively small number of included studies. These limitations highlight the need for larger randomized controlled trials, including measurements of circulating inflammatory (adipo)cytokines, to understand potential underlying pathophysiological mechanisms and to gain further insight into the direction of this association and causality (128, 129). In addition, we recommend the narrative review by Cervoni et al., that provides a general overview of behavioral weight loss treatments in migraine and obesity (130), and the review article by Razeghi Jahromi et al., that differentiates between nutritional interventions (diets) which, in general, seem to be effective in ameliorating headache/migraine (131).

Besides potential effects of obesity treatments on the (natural) course of migraine, the effect of BMI on migraine treatment is another point of debate. A prospective study was conducted in adults (aged 18–45 years), subdivided in four increasing BMI categories, treated with nortriptyline and propranolol to evaluate the effect of BMI on these preventive treatments (132). After a follow-up of 8 weeks, results showed a direct and significant influence of BMI on the mean pain duration, frequency, and severity. The latter was evaluated by the visual analog scale (VAS) and 6-point behavioral rating scale (BRS-6). These outcomes were in favor of the group of participants with a lower BMI (132). A similar pattern was observed in the AMPP study, as the use of preventive treatment was more common in the obese and morbidly obese groups with (probable) migraine. However, no association was observed between the use of acute antimigraine drugs and BMI in subjects with (probable) migraine (113).

Furthermore, vitamin D might be involved in the pathophysiology of both migraine and obesity. Indeed, next to pharmacological treatment for migraine, vitamin D has been recommended as supplementary treatment for migraine attacks (133) and obesity (134). Several studies have reported that there is a deficiency of vitamin D in patients with migraine (135–139), which may be related to the duration and frequency of migraine attacks, as well as the appearance of aura and other characteristics of migraine (e.g., phonophobia and/or photophobia); while other studies have shown normal vitamin D levels (140), or no differences (141). Regarding the relationship between migraine and obesity, it has been reported that there is no relationship between vitamin D levels and BMI and waist circumference in patients with migraine (142, 143), even after adjusting for some confounding factors (142). Although the results of the current literature are controversial, it has been suggested that supplementation with vitamin D may be beneficial in patients with migraine who have a vitamin D deficiency, which may help reduce the frequency of migraine attacks (144). Definitely, further studies should be carried out to elucidate the role of vitamin D and its relationship with migraine and obesity, considering the confounding factors (e.g., sex, age, physical activity, and sun exposure, among others).

### Possible Mechanisms Involved in the Relationship Between Migraine and Obesity

As mentioned above, migraine and obesity can be associated in different ways (see Figure 1) by sharing several factors and mechanisms, including: (i) genetic and environmental factors; (ii) the role of different neuropeptides and/or adipokines (summarized in Table 1); and (iii) the influence of central and peripheral mechanisms (see Figure 2).

#### Uni- or Bidirectional Relationship: Influence of Lifestyle

Both migraine and obesity are associated with a high social, personal, and economic impact affecting the quality life of individuals. These disorders are also related to changes in lifestyle and environmental and genetic factors. Despite the unidirectional association between migraine and obesity (i.e., obesity produces an increase in migraine attacks) (7), we could suggest that there is a bidirectional relationship (i.e., migraine may also be a risk factor for exacerbating the development of obesity) (see Figure 1) since: (i) the current prophylactic antimigraine drugs (e.g., β-adrenoceptor antagonists, antiepileptics, antidepressants or Ca^2+^ channel blockers) produce increases in body weight (123); and (ii) migraine is a disabling disorder (2), which can affect daily life and decrease the physical activity of those who suffer from migraine, while the lack of physical activity or a sedentary lifestyle is related to the development of obesity (72). As we mentioned before, weight loss after bariatric surgery may be beneficial in reducing the duration and severity of migraine attacks, and even in preventing them (128, 129). Moreover, several studies have reported that weight loss related to lifestyle sliming strategies, such as physical activity and exercise and/or sports help reduce the frequency of migraine attacks or might exert a protective effect on individuals who suffer from it (145–147). Nevertheless, the specific role of exercise in migraine is still unclear, especially when considering that both the frequency and intensity of exercise/sports can also trigger migraine attacks (146). Therefore, further studies are needed to understand the association between sports and migraine (147), keeping in mind the study limitations and confounding factors.

#### Biochemical Biomarkers

A potential mechanism to describe (in part) the association between migraine and obesity could be the role of common biochemical biomarkers in both disorders (see Table 1).

Pro-inflammatory mediators play an important role in (patho)physiological processes such as inflammation and pain. In obese individuals, there is an inflammatory state which may enhance the frequency, severity and duration of migraine attacks (7, 95). In this respect, an increase in plasma or serum levels of IL-1β, IL-6, IL-8, and TNF-α, as well as C-reactive protein in both migraineurs (with and without aura) (27–29) and in obese (30–32) individuals have been reported. Therefore, these pro-inflammatory mediators may be involved in the pathogenesis of migraine or in the development and progression of obesity.

On the other hand, the involvement of neuropeptides seems to be pivotal in eliciting the link between migraine and obesity, specifically the role of CGRP, the main neuropeptide involved in the pathophysiology of migraine (81). Activation of the trigeminovascular system results in a neurogenic inflammatory process and in the release of pro-inflammatory neuropeptides, including CGRP and substance P (79–81). In both migraine and obese individuals, both CGRP (18, 19, 21) and substance P (19, 22) levels are increased. In migraine, CGRP is involved in the development of headache and migraine-related symptoms (77), while in obesity, fat intake may promote CGRP release due to increased activity of sensory nerves in obesity (23). In addition, release of substance P is enhanced by CGRP from sensory nerves. An increase in the release of substance P, as well as CGRP after the activation of the nociceptive afferent system suggests the involvement of these neuropeptides in migraine attacks (79). Furthermore, since the inhibition of substance P promotes weight loss and may prevent the development of obesity, it has been suggested that this neuropeptide plays an important role in the onset and development of obesity (148, 149).

Finally, adipokines and sex hormones may be involved in the link between migraine and obesity. On the one hand, adipokines may contribute to the neurogenic inflammation of migraine, which has suggested a relationship between these disorders (150). In this respect, leptin, the main hormone playing an important role in the regulation of food intake and body weight, is increased in patients with migraine (i.e., migraine with and without aura) and obesity (38, 39) (Table 1). This increase is related to inflammatory processes via the release of pro-inflammatory cytokines (151) underlying migraine attacks and obesity (95). Moreover, leptin might be involved in the pathogenesis of migraine with and without aura (38). Furthermore, adiponectin, which is involved in energy homeostasis and in the metabolism of glucose and lipids, plays a role in the pathophysiology of migraine (37, 40, 150). Indeed, serum levels of adiponectin are increased in both episodic and chronic migraine (40), as well as in patients with migraine with aura (37), while in obese patients these levels are decreased (37, 41) (see Table 1). A possible explanation for understanding the role of adipokines and their relationship with migraine and obesity, may imply their ability to participate in inflammatory processes (37, 40, 151), which are characteristic of both disorders. Additionally, it has been reported that both leptin and adiponectin levels are higher in women than in men (37, 150). In view that migraine is a more prevalent disorder in women than in men (1), it can be suggested that these adipokines could be involved in sexual dimorphism in migraine. On the other hand, considering the importance of sex hormones, including estrogen and testosterone, in migraine pathophysiology (152) and the role of adipose tissue which serves as an endocrine organ by secreting sex hormones (153), the association between migraine and obesity might be (partly) attributable to sex hormones. Indeed, the relationship between migraine and obesity has been suggested to be the strongest in women of reproductive age (106, 121). Furthermore, an absence of the association is observed in men older than 55 years, while in women of this age category, abdominal obesity has been shown to be associated with a decrease in migraine prevalence (154). Yet, a comprehensive understanding of this association is hindered due to: (i) a lack of hormonal level assessments, (ii) the absence of stratification by sex in studies which found negative results on the association, (iii) the inclusion of only older individuals or only premenopausal women, and (iv) the absence of concordance between studies (121).

#### Central and Peripheral Mechanisms

The influence of both the central and peripheral nervous systems during migraine attacks as well as the development of obesity may be a potential mechanism to describe the association between these two disorders (Figure 2). Moreover, it has been reported that some lifestyle habits including physical activity, diet, alcohol consumption, and smoking can modify the activity of the central (155) and the autonomic nervous systems (156).

Next to regulation of body temperature, sleep, food, and water intake, hypothalamic activation is involved in starting, maintaining, and ending the migraine attacks (77). A dysregulation in the hypothalamic activity produced by the influence of the habits in the lifestyle such as sleep disruption (or insomnia) and/or the increase in food intake can trigger migraine attacks and obesity, respectively. Several studies have shown that individuals with sleep disorders are prone to morning headaches and, consequently, to developing migraine attacks (157, 158). Besides, obesity and obstructive sleep apnea are strongly associated (159). In this respect, patients with migraine, especially older men with chronic migraine and a high BMI, seem to have a high risk of sleep apnea, whose treatment (e.g., with an oral appliance) might be beneficial in some patients (160, 161). To date, the causal relationship between obstructive sleep apnea and migraine has not been established, although this sleep disturbance may be a trigger factor that could accelerate migraine progression (162). Moreover, it has been suggested that migraine attacks are related to chronobiological mechanisms that are involved in the triggering and onset of migraine attacks (163), which might be related to individual chronotypes (164). Indeed, several studies have investigated chronotype and migraine, suggesting that chronotype has an influence on the number and duration of migraine attacks, being more recurrent during the night and the first hours of the morning (163–165). In addition, hypothalamic dysfunction (by increasing food intake and unhealthy diets) is involved in inflammatory processes via the release of pro-inflammatory mediators relevant in both migraine and obesity (see Table 1). Therefore, we can suggest that hypothalamic dysfunction could be an important link between migraine and obesity.

Additionally, peripheral mechanisms (including both parasympathetic and sympathetic systems) have been considered important in both migraine and obesity (Figure 2). Individuals with migraine with aura have been shown to present sympathetic autonomic dysfunction when compared to individuals with migraine without aura (166), which can be related with an imbalance of sympathetic (co)transmitters (due to hypo- or overactivity), including noradrenaline, dopamine, prostaglandins, adenosine triphosphate, and adenosine (166, 167). Likewise, obesity is characterized by the presence of both sympathetic hypofunction and overactivity (7, 168). Furthermore, leptin (which is increased in both migraine and obesity), is associated with cortical spreading depression, suggesting that increases in leptin levels in obesity may induce chronic migraine (169). This might be an approach to understand the relationship between migraine and obesity.

## Association Between Migraine and Diabetes Mellitus (DM)

The possible relationship between migraine and DM remains unclear due to few existing studies (compared to those on obesity) and controversial results. Since obesity (which is a risk factor for migraine attacks) may increase the development of DM (mainly type 2 DM), a relationship between migraine and DM can be hypothesized. However, clinical- and population-based studies have reported that prevalence of migraine in diabetic individuals is lower (54–57), similar (58–60) or higher (61) compared with non-diabetic individuals.

### Clinical and Epidemiological Evidence

In a case-control study by Bigal et al., which was part of the aforementioned AMPP study, cross-sectional analyses were performed to compare the likelihood of self-reported diabetes between individuals with migraine and individuals who remained free from headache. Overall, it was shown that individuals with migraine were more likely to have diabetes compared to individuals without migraine (12.6 vs. 9.4%, odds ratio 1.4, 95% CI [1.2–1.6]) (15). Within the same study, no difference in the frequency of diabetes was found between adults suffering from chronic migraine and episodic migraine (114).

These results are not in line with data obtained from other studies. A Norwegian cohort study, consisting of males and females below 80 years, showed that both type 1 and type 2 DM were significantly associated with a decreased risk of migraine (risk ratio 0.74, 95% CI [0.61-0.89] and risk ratio 0.86, 95% CI [0.80–0.92], respectively) (170). Moreover, to investigate the association between migraine status and incident type 2 DM, multivariable-adjusted analysis within the large prospective Women's Health Study were performed. It was found that women with any history of migraine had a lower prevalence of having diabetes at baseline (prevalence odds ratio 0.79, 95% [0.67–0.94]). In addition, researchers found no association between any history of migraine and incident type 2 DM (60). Besides, Fagherazzi et al. investigated the association—including the temporality of this potential association—between migraine and (pharmacologically treated) type 2 DM within a French prospective cohort study, the E3N study. A lower risk of type 2 DM was observed in women suffering from active migraine. Secondary analyses showed a linear decrease of the migraine prevalence prior to the diabetes diagnosis, subsequently followed by a plateau after the diabetes diagnosis (62). In contrast, studies by López-de-Andrés et al. and Haghighi et al. found no significant differences in migraine prevalence between individuals with and without diabetes (12, 59).

These conflicting results might be attributable to methodological limitations. Not all studies differentiated between type 1 and type 2 DM. Indeed, Hagen et al. found an inverse relationship between type 1 diabetes and migraine in the Nord-Trøndelag Health Study (171). Also, obesity has been shown to be related to both type 1 and type 2 DM (44) and migraine (7, 50); it might therefore serve as a confounding variable when examining the relationship between migraine and DM, as discussed below in more detail. In addition, age might be an important effect modifier in the association between type 2 DM and migraine, demonstrating an inverse association (lower hazard risk for migraine in patients with diabetes) in the elderly (170). As various studies included specific age categories, no ultimate conclusions can be drawn yet. An older study by Burn et al. showed that the prevalence of migraine was lower in diabetic patients in all age groups and in both sexes (54).

We highly recommend a recently published systematic literature review by Hosseinpour et al. (172) for a comprehensive overview of studies on the association between migraine and DM.

#### Lifestyle Interventions and Treatments in Migraine and DM

Insulin resistance (IR) has been proposed as an underlying pathophysiological mechanism involved in the association between migraine and diabetes (173), although not all studies found this link (174). Migraine patients have been reported to have increased insulin levels compared to controls (175, 176). Increased fasting levels of neuropeptide Y (177), leptin and glucagon-like 2 peptide (176) in migraine patients have been suggested to play a role. In addition, IR has been correlated with migraine characteristics, including the duration of attacks (16).

Therefore, IR-lowering compounds have been hypothesized to lower the headache frequency. Indeed, Cavestro et al. performed an open-label cohort study to investigate the effects of an IR-lowering drug, alpha-lipoic acid, in migraine patients (178). They found a significant decrease in the number of migraine attacks and treatment days, probably due to its antioxidant effects, as these reductions were not related to changes in laboratory assessments such as quantitative insulin sensitivity check index and Stumvoll index (both are markers of insulin sensitivity), glucose, and insulin. Also, two case-reports described female chronic migraine sufferers, who showed a profound improvement of their symptoms after insulin administration (179).

### Possible Mechanisms Involved in the Relationship Between Migraine and DM

Despite the scarce evidence and the contradictory results that exist about the relationship between migraine and DM, we can hypothesize some mechanisms involved in an inverse relationship between these disorders (see Figure 2). However, the protective role of DM in migraine attacks remains unclear.

#### Lifestyle Habits

Both migraine and obesity can be triggered by the influence of lifestyle habits, as well as environmental and genetic factors (Figure 1). Since obesity is a risk factor for migraine (7, 50) and for both type 1 and type 2 DM (44–46), it could be interpreted that obesity is a confounder factor. However, besides obesity, several authors have reported that lifestyle habits and comorbidities may explain the association between migraine and DM (57, 59, 60). In this respect, lifestyle habits including sedentary life, smoking and alcoholism, as well as conditions such as pain, respiratory disease, and mental disorders in diabetic individuals are related with a higher prevalence in migraine attacks (12). However, further research is needed to establish a causal association between DM and migraine.

#### Biochemical Biomarkers

Relevant pro-inflammatory markers in migraine have been reported to be elevated during DM as well (Table 1). Inflammatory processes are implicated in both the onset and progression of type 1 and type 2 DM and their complications (180), probably due to poor control of glucose concentrations (33). In this respect, C-reactive protein, IL-1β, IL-6, IL-8, and TNF-α are increased in diabetic individuals (32–36). We can hypothesize that this could be a key to understanding the possible association between DM and migraine. However, we cannot categorically exclude that inflammatory processes during DM are partly also caused by obesity, considering their comorbidity.

In contrast, neuropeptides (which are increased in migraine and obesity) are decreased in DM (see Table 1). In diabetic individuals, CGRP and substance P levels are decreased (21, 22, 24). Moreover, it has been reported that in an experimental model of type 1 diabetes there is a decrease in or an impairment of CGRPergic sensory nerves (181, 182). In addition, it is well-known that CGRP is a potent vasodilator which is associated with glucose metabolism. In this respect, CGRP-induced vasodilation and nociceptive effects are impaired after the onset of diabetes, which can explain the reduced prevalence of migraine attacks (62). Certainly, CGRP exerts a hyperglycaemic effect (183), but the evidence has shown that diabetic individuals presented lower CGRP levels (see Table 1). Since hypoglycemia is responsible for triggering and exacerbating migraine (59) (probably due to CGRP-induced hyperinsulinemia), this could suggest an inverse relationship between migraine and DM.

Regarding the role of adipokines, leptin is increased in diabetic patients (39), as well as in patients with migraine and obese individuals (Table 1). This adipokine is involved in obesity and IR, two conditions associated with the development of DM (184). Therefore, the relationship between diabetes and leptin could be based on inflammatory processes that exist during DM. Moreover, adiponectin levels are increased in type 1 DM, particularly in patients with poor diabetes control (42). In contrast, patients with type 2 DM show low levels of adiponectin, when compared to non-diabetic patients (43) (Table 1). In this respect, it has been suggested that: (i) adiponectin has anti-diabetic and anti-inflammatory properties (185); and (ii) a decrease in its circulating levels may predict the risk of type 2 DM and/or obesity (186). However, there is no evidence on the role of these adipokines in migraine individuals with DM.

#### Central and Peripheral Mechanisms

During DM, metabolic changes take place that are influenced by the central and peripheral nervous systems, specifically by the autonomic nervous system. Potential mechanisms that may be involved in the relationship between migraine and DM, which are also related with lifestyle habits, are depicted in Figure 2. As described above, lifestyle habits produce modifications in the activity of the central (92) and the autonomic nervous systems (93), which can independently trigger migraine attacks or trigger DM. However, the relationship between these two conditions remains unclear.

The hypothalamus is involved in regulating glucose metabolism. Changes in glucose concentrations are sensed by hypothalamic and brainstem activation (187). In this respect, hypoglycemia-regulated insulin in the hypothalamus has been associated with migraine (59). In fact, it has been suggested that symptoms during hypoglycemia and migraine are associated (i.e., dizziness, nausea, fatigue, visual dysfunction, etc.) (77), and hypothalamic activation during migraine could represent a mechanism to understand the premonitory symptoms during migraine to an adaptive response to glucose changes sensed by the hypothalamus (77, 188).

Additionally, the sympathetic nervous system and autonomic dysfunction, due to a lack of physical activity, play an important role in the development of DM and its complications (101). Moreover, DM is related to peripheral catecholamines abnormalities. In this respect, an experimental model of type 1 diabetes suggested a differential role of the monoamines receptors involved in the prejunctional modulation of the sympathetic vasopressor and cardioaccelerator outflows (189, 190), which can be related to autonomic complications during diabetes. These catecholamines are also involved in the sympathetic dysfunction in migraine and obesity, as mentioned above. Thus, we can suggest that further studies should analyze whether sympathetic dysfunction is a mechanism underlying the relationship between migraine and DM, even considering that a proper lifestyle can improve autonomic dysfunction in both migraine and diabetes.

## Other Metabolic Disorders

Besides obesity and DM, IR and metabolic syndrome have been associated with migraine. IR, a metabolic condition characterized by a decreased response to insulin (11), plays an important role in the development of metabolic syndrome, which is linked to obesity, type 2 DM, hypertension, and (cardio)vascular risk factors (e.g., coronary disease, ischemia, stroke) (10, 11), all of which are risk factors for migraine as well. Considering that IR has been strongly considered as a risk factor for (cardio)vascular disease, its role might be relevant in patients with migraine (191).

Several studies have reported an association between migraine, IR and/or metabolic syndrome (173, 175, 176, 192–195). In this respect, Rainero et al. (173) evaluated insulin sensitivity in non-obese, non-diabetic and normotensive young patients with migraine. In this study, insulin sensitivity (ISI-Stumwoll index) and oral glucose insulin sensitivity (OGIS-180 index) were significantly altered in migraine patients when compared with non-migraine patients (0.28 ± 0.12 vs. 0.18 ± 0.09; and 455.4 ± 59 vs. 515.7 ± 088.2, *p* < 0.01; respectively) (173). Similar results were reported by Cavestro et al. (175), who observed that in patients with migraine insulin concentrations were significantly higher, a finding suggesting that insulin sensitivity is impaired in migraine (175). Moreover, it has been observed that the 1-year migraine prevalence in metabolic syndrome is 11.9% in men and 22.5% in women, who also presented increased values of BMI, waist circumference and a high percentage of DM (considered as metabolic syndrome components) (192). These results suggest that IR might underlie a common pathogenesis of migraine and metabolic syndrome (192). In addition, there are some clinical studies that confirm the relationship between IR and episodic or chronic migraine (176, 194, 195). In contrast, only a few studies failed to support the association of migraine with IR (16, 191).

## Future Implications

In this review, we aimed to provide a comprehensive overview of the relationship between migraine and two metabolic comorbidities with high worldwide prevalence, namely, obesity and DM. Despite several epidemiological studies that have hypothesized a relationship, we consider that the mechanisms are not completely established. Moreover, since these three disorders are related with lifestyle habits, it is of interest to understand the underlying (patho)physiological mechanisms in order to improve the quality of life of the people who suffer from them. Consequently, several issues need to be addressed.

Firstly, is obesity a risk factor for migraine attacks or is migraine a risk factor for obesity? Several authors have suggested a unidirectional association between obesity and migraine (7, 47, 48), considering obesity as a risk factor that can trigger migraine attacks. However, lifestyle habits and environmental factors such as overmedication, sedentary life, or psychological disorders during migraine attacks can produce an increase in body weight or obesity (73, 107); hence, we suggest a bidirectional mechanism between migraine and obesity (Figure 1). Regarding the association between migraine and DM, results remain unclear and the existing association seems to be inverse, considering DM as a protective factor against migraine attacks (13, 57). Both migraine and diabetes (as well as obesity) share lifestyle-related factors that are involved in their etiology and pathophysiology (see Figure 1). However, the mechanisms involved are not clear enough. Moreover, although obesity is a trigger for DM (44–46), it does not ensure that DM can display the same relationship with migraine.

Secondly, clinical and epidemiological studies have tried to explain the association between migraine and both obesity and DM (*see section on Clinical and Epidemiological Evidence*). Nevertheless, the strength of this association differs across the literature. As in most studies, there are limitations and uncontrolled variables which are considered as “confounding factors”; these may include age, sex, marital and socio-economic status, depressive symptoms, pharmacotherapy, and problems of misclassification of migraine and obesity, as well as the diagnosis of DM (12, 13), leading to potential bias. However, controlling for these confounders might take away potential associations between migraine and obesity and effect-modifiers, including attack frequency, age, and sex, but also perceived stress (196). Remarkably, studies on the association between migraine and DM have identified a more limited amount of effect-modifiers, mainly due to the lack of stratification of their results (e.g., sex-specific estimates) (172). Yet, obesity should be considered as an effect-modifier in the association between migraine and DM (or IR) (194).

In addition, shared comorbidities such as depression and anxiety have been reported to have modifying effects on the strength of the association between obesity and migraine frequency as well as headache-related disability. Indeed, obese subjects with comorbid depression or anxiety seemed to be more likely to suffer from higher headache frequency compared to subjects with a normal weight without depression or anxiety (197). Furthermore, as mentioned before, a variety of migraine prophylactics (including amitriptyline, fluoxetine, nortriptyline, paroxetine, divalproex sodium, flunarizine, and methysergide) are associated with weight gain as a side-effect (123). However, as noted by Ornello et al. (106), current studies did not take into account the effects of weight gain due to these prophylactics. Therefore, the use of these medications might be an overlooked confounding factor in the probable bidirectional relationship between migraines and obesity. A similar restriction applies to studies on the association between DM and migraine, which lack data on drug use that not only influence weight, but also insulin sensitivity (172). Another cause of the observed variability might be related to the fact that not all articles used the same indices of obesity. Indeed, the use of different measures, including BMI, body fat percentage, the waist-hip ratio, and the body fat percentage, might lead to inconsistencies in these results. Besides, using BMI has the disadvantage that it does not distinguish between different body compositions, including fat mass and muscle mass or abdominal and peripheral fat distribution. Age and sex are important determinants of body fat distribution and migraine prevalence, which even further underline the importance of taking these effect-modifiers into account (110).

Thirdly, it is important to emphasize that an improvement in lifestyle can reduce the frequency of migraine attacks or the DM- and obesity-related complications, probably by affecting central and peripheral mechanisms. Moreover, it has been suggested that preventive antimigraine drugs can improve metabolic functioning (77). Nevertheless, to the best of our knowledge, there is no available treatment yet for a synergistic effect to improve the quality of life of people suffering from migraine and obesity, or migraine and DM. Since CGRP seems to be pivotal in the pathophysiology of migraine, and also in the regulation of energy metabolism, it is tempting to speculate whether blocking CGRP or its receptor would be a therapeutic strategy for these disorders. In this respect, an important aspect to consider is the association between cardiovascular diseases (including cerebrovascular diseases) and the three disorders: migraine (particularly with aura) (14, 15, 198), obesity (aside from the obesity paradox (i.e., the potentially protective effect of obesity found in epidemiological studies probably due to bias) (199, 200), and DM (201). These disorders have been described to be independent cardiovascular risk factors. Certainly, a recent study published by Kurth et al. puts these risk factors into perspective (202). Compared to migraine with aura, DM (and current smoking) are associated with higher incidence rates of major cardiovascular disease. However, migraine with aura has a stronger association with major cardiovascular diseases compared to obesity (or unfavorable lipid levels). Therefore, blocking the CGRPergic system (in the long-term) might not be safe in terms of cardiovascular safety (203, 204), especially for obese and diabetic individuals. We suggest that both basic experimental science and clinical studies (in migraine patients with metabolic comorbidities such as obesity and DM) could be relevant to establish the role of CGRP as a link between migraine and metabolic disorders. Therefore, CGRP might be a potential therapeutic target for metabolic disorders as well.

Finally, randomized controlled trials (including dietary interventions in obese women with and without migraine, and preventive medication in migraine patients with and without obesity), as well as the application of Mendelian randomization in observational studies are needed to establish the causality of the association between migraine and obesity as well as DM. Due to the controversial results (probably due to methodological limitations), it seems that in cross sectional studies it is not possible to determine the causality of this association with certainty. Therefore, we suggest that future research should consider different variables (e.g., sex hormones, reproductive age, as well as a correct diagnosis for migraine or obesity) and biochemical biomarker measurements (including measurements of CGRP and pro-inflammatory mediators); these, in turn, may help clarify the relationship between these disorders, even considering other possible comorbidities or risk factors that may develop during the progression of migraine, obesity and DM, such as cardiovascular complications. In addition, future research is needed on the role of treatment for overweight and obesity, as well as antidiabetic therapies on disease progression in migraine patients and vice versa, taking the limited available literature—especially in diabetics—into account.

## Conclusion

Lifestyle habits can provide an essential link between migraine and obesity, or between migraine and diabetes. Based on the current evidence, we propose that there is a bidirectional relationship between migraine and obesity, taking into account that obesity is a risk factor for migraine attacks, and that migraine (or its pharmacological treatment) may be a risk factor for the development of obesity. In addition, shared factors between migraine and DM could help understand this (inverse) relationship. Therefore, we suggest that the mechanisms to explain the association between migraine and these metabolic disorders include: (i) lifestyle, genetic and environmental factors; (ii) biochemical biomarkers that are relevant in the pathophysiology of migraine (e.g., CGRP and pro-inflammatory markers); and (ii) central and peripheral mechanisms (which can be modified by the influence of lifestyle habits). However, future research is required for considering different variables (e.g., sex hormones, reproductive age, and metabolic comorbidities) to identify the underlying mechanisms and to establish the link between migraine and metabolic disorders. Ultimately, this is needed to find a potential therapeutic target to control and improve the quality of life of the people who suffer from these diseases.

## Author Contributions

ER-M and AM organized and led the review. The content of this article was made by consensus of all the authors. All authors listed have made a substantial, direct and intellectual contribution to the work, and approved it for publication.

## Conflict of Interest

The authors declare that the research was conducted in the absence of any commercial or financial relationships that could be construed as a potential conflict of interest.
