# Supplementary material for: Metabolic Aspects of Migraine: Association With Obesity and Diabetes Mellitus
Source: Front Neurol. 2021 Jun 9;12:686398. doi: 10.3389/fneur.2021.686398 (PMC8219973; doi:10.3389/fneur.2021.686398)
Supplement: Supplementary file 1 [file Data_Sheet_1.pdf]

Appendix 1.

Search strategy narrative review performed on 4th of January 2021

Database searched	Via	Years of coverage	Records	Records after duplicates removed
Embase	Embase.com	1971 - Present	274	269
Medline ALL	Ovid	1946 - Present	188	30
Web of Science Core Collection	Web of Knowledge	1975 - Present	114	6
Cochrane Central Register of Controlled Trials	Wiley	1992 - Present	25	17
Other sources: Google Scholar (50 top-ranked)			19	14
**Total**			**620**	**336**

Embase

('migraine'/exp/mj OR 'primary headache'/exp/mj OR ('calcitonin gene related peptide'/mj AND ('migraine'/exp OR 'primary headache'/exp)) OR ((((CGRP OR CGRPs OR calcitonin-gene-related-peptide*):ti,kw AND (migrain* OR primary-headache*)):ti,ab,kw) OR (migrain* OR primary-headache*):ti,kw)) AND ('obesity'/exp/mj OR 'diabetes mellitus'/exp/mj OR 'hyperglycemia'/de/mj OR 'insulin resistance'/de/mj OR 'insulin metabolism'/exp/mj OR 'body mass'/mj OR 'metabolic syndrome X'/exp/mj OR (obesit* OR obese OR adiposit* OR adipositas OR over-weight* OR overweight* OR ((excess* OR high*) NEAR/3 (body) NEAR/3 (weight*)) OR ((diabet*) NOT (insipid*)) OR diabetic OR NIDDM OR T2DM OR hyperglycem* OR ((metaboli* OR metaboloendocrin*) NEAR/3 (syndrom*)) OR insulin-resistan* OR insulin-metabolism* OR ((impair*) NEAR/3 (glycemia* OR glycaemia*)) OR body-mass* OR BMI):ti,kw) NOT ([Conference Abstract]/lim) NOT ('case report'/de OR (case-report*):ti) AND [ENGLISH]/lim

Medline

(*Migraine without Aura/ OR *Headache Disorders, Primary/ OR (*Calcitonin Gene-Related Peptide/ AND (Migraine without Aura/ OR Headache Disorders, Primary/)) OR ((((CGRP OR CGRPs OR calcitonin-gene-related-peptide*).ti. AND (migrain* OR primary-headache*).ti,ab.)) OR (migrain* OR primary-headache*).ti,kf.)) AND (exp *Obesity/ OR exp *Diabetes Mellitus/ OR exp *Hyperglycemia/ OR exp *Insulin Resistance/ OR *Body Mass Index/ OR (obesit* OR obese OR adiposit* OR adipositas OR over-weight* OR overweight* OR ((excess* OR high*) ADJ3 (body) ADJ3 (weight*)) OR ((diabet*) NOT (insipid*)) OR diabetic OR NIDDM OR T2DM OR hyperglycem* OR ((metaboli* OR metaboloendocrin*) ADJ3 (syndrom*)) OR insulin-resistan* OR insulin-metabolism* OR ((impair*) ADJ3 (glycemia* OR glycaemia*)) OR body-mass* OR BMI).ti,kf.) NOT (news OR congres* OR abstract* OR book* OR chapter* OR dissertation abstract*).pt. NOT (Case Reports/ OR (case-report*).ti.) AND english.la.

Cochrane

(((((CGRP OR CGRPs OR calcitonin NEXT gene NEXT related NEXT peptide*):ti AND (migrain* OR primary NEXT headache*)):ti,ab) OR (migrain* OR primary NEXT headache*):ti)) AND ((obesit* OR obese OR adiposit* OR adipositas OR over NEXT weight* OR overweight* OR ((excess* OR high*) NEAR/3 (body) NEAR/3 (weight*)) OR ((diabet*) NOT (insipid*)) OR diabetic OR NIDDM OR T2DM OR hyperglycem* OR ((metaboli* OR metaboloendocrin*) NEAR/3 (syndrom*)) OR insulin NEXT resistan* OR insulin NEXT metabolism* OR ((impair*) NEAR/3 (glycemia* OR glycaemia*)) OR body NEXT mass* OR BMI):ti)

Web of Science

(((((TI=(CGRP OR CGRPs OR calcitonin-gene-related-peptide*) AND TS=(migrain* OR primary-headache*))) OR TI=(migrain* OR primary-headache*))) AND TI=((obesit* OR obese OR adiposit* OR adipositas OR over-weight* OR overweight* OR ((excess* OR high*) NEAR/2 (body) NEAR/2 (weight*)) OR ((diabet*) NOT (insipid*)) OR diabetic OR NIDDM OR T2DM OR hyperglycem* OR ((metaboli* OR metaboloendocrin*) NEAR/2 (syndrom*)) OR insulin-resistan* OR insulin-metabolism* OR ((impair*) NEAR/2 (glycemia* OR glycaemia*)) OR body-mass* OR BMI))) AND DT=(Article OR Review OR Letter OR Early Access) AND LA=(English)

Google Scholar (search in title words only)

migraine|'primary headache' obese|obesity|overweight|diabetes|diabetic|'metabolic syndrome'|hyperglycemia|BMI|'body mass'|adiposity|adipositas|T2DM|NIDDM
